# Supplementary material for: Socio‐Demographic Variation in Adherence to the Eatwell Guide Within the UK Biobank Prospective Cohort Study
Source: Nutr Bull. 2026 May 18;51(2):258–68. doi: 10.1111/nbu.70055 (PMC13254699; doi:10.1111/nbu.70055)
Supplement: Supplementary file 1 — Table S1: List of individual foods which contributed to each food group and the EWG scoring methodology. Table S2: Characteristics of the analytical sample versus rest of cohort. Table S3: Starchy carbohydrate intake by socio‐demographic factors. Table S4: Wholegrain intake by socio‐demographic factors. Table S5: Red and processed meat intake by socio‐demographic factors. Table S6: Fish intake by socio‐demographic factors. Table S7: White meat intake by socio‐demographic factors. Table S8: Fruit and vegetable intake by socio‐demographic factors. Table S9: Dairy intake by socio‐demographic factors. Table S10: Beans and pulses intake by socio‐demographic factors. Table S11: Nuts intake by socio‐demographic factors. Table S12: Egg intake by socio‐demographic factors. Table S13: Discretionary foods intake by socio‐demographic factors. Table S14: Fluid intake by socio‐demographic factors. Table S15: Proportion of participants achieving adherence to individual components (i.e., 5 points) of the Eatwell Guide. Table S16: Sensitivity analysis demonstrating adherence to the Eatwell Guide by socio‐demographic characteristics, only including those participants who completed a minimum of two dietary reports. Table S17: Sensitivity analysis demonstrating adherence to the Eatwell Guide by socio‐demographic characteristics, excluding dietary reports with extreme energy intakes. Table S18: Sensitivity analysis of EWG adherence score by socio‐demographic factors when sequentially removing one component of EWG. [file NBU-51-258-s001.docx]

**Table S1** – List of individual foods which contributed to each food group and the EWG scoring methodology

| **Food component** | **Contributing foods from the Oxford WebQ** | **Average consumption required (servings/day)** | **EWG Score** | **Scoring development details** |
| --- | --- | --- | --- | --- |
| **Fruit and vegetables** | Stewed fruit (104410), Prune (104420), Dried fruit (104430), Mixed fruit (104440), Apple (104450), Banana (104460), Berry (104470), Cherry (104480), Grapefruit (104490), Grape (104500), Mango (104510), Melon (104520), Orange (104530), Satsuma (104540), Peach nectarine (104550), Pear (104560), Pineapple (104570), Plum (104580), Other fruit (104590), Orange juice (100190), Grapefruit juice (100200), Pure fruit vegetable juice (100210), Fruit smoothie (100220), Mixed veg (104060), Veg pieces (104070), Coleslaw (104080), Side salad (104090), Avocado (104100), Beetroot (104130), Broccoli (104140), Butternut squash (104150), Cabbage kale (104160), Carrot (104170), Cauliflower (104180), Celery (104190), Courgette (104200), Cucumber (104210), Garlic (104220), Leek (104230), Lettuce (104240), Mushroom (104250), Onion (104260), Parsnip (104270), Sweet pepper (104290), Spinach (104300), Sprouts (104310), Sweetcorn (104320), Fresh tomato (104340), Tinned tomato (104350), Turnip swede (104360), Watercress (104370), Other veg (104380), Olive (102490) | From 0 to < 2.5 | 0 | Specific quantitative details provided in the Eatwell Guide |
|  |  | From 2.5 to < 3.125 | 1 |  |
|  |  | From 3.125 to < 3.75 | 2 |  |
|  |  | From 3.75 to < 4.375 | 3 |  |
|  |  | From 4.375 to < 5 | 4 |  |
|  |  | ≥5 | 5 |  |
| **Starchy Carbohydrates** | White pasta (102710), Wholemeal pasta (102720), White rice (102730), Brown rice (102740), Snackpot (102760), Couscous (102770), Other grain (102780), Sliced bread (100950), Baguette (101020), Bap (101090), Bread roll (101160), Naan bread (101230), Garlic bread (101240), Crispbread (101250), Oatcake (101260), Other bread (101270), Porridge (100770), Muesli (100800), Oat crunch (100810), Plain cereal (100830), Bran cereal (100840), Wholewheat cereal (100850), Other cereal (100860), Fried potatoes (104020), Boiled baked potatoes (104030), Mashed potato (104050), Sweet potato (104330) | **Men**  < 2.5  **Women**  < 2 | 0 | Sex-specific energy intake recommendations to estimate the proportion of calories allocated to each food group based on the relative size of the segment in the EWG plate.  These calorie estimates were converted to grams and then to standard serving sizes (based on average servings of specific foods). |
|  |  | **Men**  From 2.5 to < 3.125  **Women**  From 2 to < 2.5 | 1 |  |
|  |  | **Men**  From 3.125 to < 3.75  **Women**  From 2.5 to < 3 | 2 |  |
|  |  | **Men**  From 3.75 to < 4.375  **Women**  From 3 to < 3.5 | 3 |  |
|  |  | **Men**  From 4.375 to < 5  **Women**  From 3.5 to < 4 | 4 |  |
|  |  | **Men**  ≥ 5  **Women**  ≥ 4 | 5 |  |
| **Wholegrains** | Wholemeal pasta (102720), Brown rice (102740), Sliced bread (100950), Type of bread (20091), Baguette (101020), Type of baguette (20092), Bap (101090), Type of bap (20093), Bread roll (101160), Type of bread roll (20094), Oatcake (101260), Porridge (100770), Muesli (100800), Bran cereal (100840), Wholewheat cereal (100850), Other grain (102780) | From 0 to < 1.5 | 0 | Due to the lack of specific quantitative guidance in the Eatwell Guide, these were obtained from a relevant expert organisation (British Dietetic Association) |
|  |  | From 1.5 to < 1.875 | 1 |  |
|  |  | From 1.875 to < 2.25 | 2 |  |
|  |  | From 2.25 to < 2.625 | 3 |  |
|  |  | From 2.625 to < 3 | 4 |  |
|  |  | ≥ 3 | 5 |  |
| **Beans/pulses** | Pea (104280), Green beans (104120), Broad beans (104110), Baked beans (104000), Pulses (104010), Tofu (103270) | From 0 to < 0.21 | 0 | In the absence of specific quantitative recommendations in the EWG and associated modelling documents, we adapted scoring from the MEDAS (Mediterranean Diet Adherence Score), which aligns broadly with EWG principles. |
|  |  | From 0.21 to < 0.27 | 1 |  |
|  |  | From 0.27 to < 0.32 | 2 |  |
|  |  | From 0.32 to < 0.38 | 3 |  |
|  |  | From 0.38 to < 0.43 | 4 |  |
|  |  | ≥ 0.43 | 5 |  |
| **Fish^a^** | Tinned tuna (103150), Oily fish (103160), Breaded fish (103170), Battered fish (103180), White fish (103190), Prawns (103200), Lobster crab (103210), Shellfish (103220), Other fish (103230) | **Fish**  From 0 to < 0.14  **Oily fish**  From 0 to < 0.07 | 0 | Specific quantitative details provided in the Eatwell Guide |
|  |  | **Fish**  From 0.14 to < 0.18  **Oily fish**  From 0.07 to < 0.09 | 1 |  |
|  |  | **Fish**  From 0.18 to < 0.21  **Oily fish**  From 0.09 to < 0.11 | 2 |  |
|  |  | **Fish**  From 0.21 to < 0.25  **Oily fish**  From 0.11 to < 0.13 | 3 |  |
|  |  | **Fish**  From 0.25 to < 0.29  **Oily fish**  From 0.13 to < 0.14 | 4 |  |
|  |  | **Fish**  ≥ 0.29  **Oily fish**  ≥ 0.14 | 5 |  |
| **Poultry** | Poultry (103060), Breaded poultry (103050) | From 0 to < 0.07 | 0 | We used the Eatwell guide supplementary modelling documentation “From plate to Guide: What, why and how for the Eatwell model” (Appendix 6). This documentation provided recommended intake for poultry in which we could convert into daily servings. |
|  |  | From 0.07 to < 0.09 | 1 |  |
|  |  | From 0.09 to < 0.11 | 2 |  |
|  |  | From 0.11 to < 0.13 | 3 |  |
|  |  | From 0.13 to < 0.14 | 4 |  |
|  |  | ≥ 0.14 | 5 |  |
| **Nuts** | Unsalted nuts (102440), salted nuts (102430), unsalted peanuts (102420), salted peanuts (102410) | From 0 to < 0.07 | 0 | As above but for nuts |
|  |  | From 0.07 to < 0.09 | 1 |  |
|  |  | From 0.09 to < 0.11 | 2 |  |
|  |  | From 0.11 to < 0.13 | 3 |  |
|  |  | From 0.13 to < 0.14 | 4 |  |
|  |  | ≥ 0.14 | 5 |  |
| **Eggs** | Whole egg (102940), Omelette (102950), Egg sandwiches (102960), Scotch egg (102970), Other egg (102980) | From 0 to < 0.07 | 0 | As above but for eggs |
|  |  | From 0.07 to < 0.09 | 1 |  |
|  |  | From 0.09 to < 0.11 | 2 |  |
|  |  | From 0.11 to < 0.13 | 3 |  |
|  |  | From 0.13 to < 0.14 | 4 |  |
|  |  | ≥ 0.14 | 5 |  |
| **Red and processed meat** | Bacon (103070), Ham (103080), Liver (103090), Sausage (103010), Beef (103020), Pork (103030), Lamb (103040) | ≥ 1.5 | 0 | Lack of specific quantitative guidance within the EWG itself. However, other government recommendations highlight that intake should be limited to <70g per day (equivalent to 1 serving) (SACN, 2010). This aligns with other EWG research in the area (Scheelbeek et al., 2020) |
|  |  | From 1.375 to < 1.5 | 1 |  |
|  |  | From 1.25 to < 1.375 | 2 |  |
|  |  | From 1.12 To < 1.25 | 3 |  |
|  |  | From 1 to < 1.12 | 4 |  |
|  |  | < 1 | 5 |  |
| **Dairy** | Yogurt (102090), Low fat hard cheese (102810), Hard cheese (102820), Low fat cheese spread (102850), Cheese spread (102860), Soft cheese (102830), Goat cheese (102900), Blue cheese (102840), Feta (102880), Mozzarella (102890), Other cheese (102910), Cottage cheese (102870), Milk (100520), Flavoured milk (100530), Added milk instant coffee (100260), Added milk filtered coffee (100280), Added milk espresso (100320), Added milk other coffee (100350), Added milk standard tea (100460), Added milk rooibos tea (100480), Instant coffee (100250), Filtered coffee (100270), Espresso (100310), Other coffee (100330), Standard tea (100400), Rooibos tea (100410), Dairy smoothie (100230), Latte (100300), Cappuccino (100290), Type of milk used (100920), Added milk cereal (100890), Porridge (100770), Muesli (100800), Bran cereal (100840), Wholewheat cereal (100850), Oat crunch (100810), Plain cereal (100830), Sweetened cereal (100820), Other cereal (100860) | ≥ 3 | 0 | We used the Eatwell guide supplementary modelling documentation “From plate to Guide: What, why and how for the Eatwell model” (Appendix 6), as well as replicated what had been done in previous studies (Fadnes et al., 2023) |
|  |  | From 2.75 to < 3 | 1 |  |
|  |  | From 2.5 to < 2.75 | 2 |  |
|  |  | From 2.25 to < 2.5 | 3 |  |
|  |  | From 2 to < 2.25 | 4 |  |
|  |  | < 2 | 5 |  |
| **Discretionary foods** | Chocolate biscuit (102350), Chocolate covered biscuit (102340), Chocolate bar (102260), Chocolate sweets (102310), Chocolate raisins (102300), Dark chocolate (102290), Milk chocolate (102280), White chocolate (102270), Sweet biscuits (102360), Cakes (102190), Cheesecake (102220), Doughnut (102200), Fruitcake (102180), Danish pastry (102060), Sponge pudding (102210), Milk based pudding (102140), Other milk pudding (102150), Other desert (102230), Soya desert (102170), Sweets (102330), Diet sweets (102320), Other sweets (102380), Ice cream (102120), Fizzy drinks (100170), Squash (100180), Sugar added to tea (100490), Sugar added to coffee (100370), Sugar added to cereal (100900), Hot chocolate (100550), Pancake (102010), Scotch pancake (102020), Croissant (102050), Scone (102070), Crisp (102460), Cereal bar (102370) | ≥ 1.5 | 0 | EWG recommends that these foods are not needed in our diet and intake should be limited. This has been interpreted as less than 1 serving per day |
|  |  | From 1.375 to < 1.5 | 1 |  |
|  |  | From 1.25 to < 1.375 | 2 |  |
|  |  | From 1.12 to < 1.25 | 3 |  |
|  |  | From 1 to < 1.12 | 4 |  |
|  |  | < 1 | 5 |  |
| **Fluid** | Drinking water (100150), Instant coffee (100250), Filtered coffee (100270), Espresso (100310), Cappuccino (100290), Latte (100300), Other coffee type (100330), Decaffeinated coffee (100360), Standard tea (100400), Rooibos tea (100410), Green tea (100420), Herbal tea (100430), Other tea (100440), Decaffeinated tea (100470), Low calorie drink (100160), Squash (100180), Orange juice (100190), Grapefruit juice (100200), Pure fruit vegetable juice (100210), Fruit smoothie (100220), Dairy smoothie (100230), Type of milk used (100920), Milk (100520), Flavoured milk (100530) | From 0 to < 3 | 0 | Specific quantitative guidance included in the EWG |
|  |  | From 3 to < 3.75 | 1 |  |
|  |  | From 3.75 to < 4.5 | 2 |  |
|  |  | From 4.5 to < 5.25 | 3 |  |
|  |  | From 5.25 to < 6 | 4 |  |
|  |  | ≥ 6 | 5 |  |

a; The final EWG score for fish was created by averaging the EWG score for oily fish and the EWG score for fish overall.

To address the lack of specific quantitative guidance within the Eatwell Guide, three approaches to estimate recommended portion sizes per day for different food groups were employed:

1. For foods with visual/quantitative guidance in the Eatwell Guide (e.g., starchy carbohydrates), we used sex-specific energy intake recommendations to estimate the proportion of calories allocated to each food group based on the relative size of the segment in the EWG plate. These calorie estimates were converted to grams and then to standard serving sizes (based on average servings of specific foods).
2. For foods with qualitative guidance we used the Eatwell guide supplementary modelling documentation *“From plate to Guide: What, why and how for the Eatwell model”* (Appendix 6). This documentation provided recommended intake for specific foods (e.g. nuts, white meat), in which we could convert into daily servings. In the absence of this information and where no specific guidance exists (e.g. beans and pulses), we adapted scoring from the MEDAS (Mediterranean Diet Adherence Score), which aligns broadly with EWG principles.
3. In the absence of specific guidance within the EWG or its modelling documentation, we adopted expert consensus from the wider literature. An example of this is for wholegrains, where research and practical guidelines (e.g., by the British Dietetics Association amongst others) recommends consuming three servings of wholegrains per day for adults.

In some cases, (e.g. dairy) a combination of methods (modelling documentation and expert consensus) were used to identify serving numbers which most closely align with the Eatwell Guide recommendation.

**Table S2. Characteristics of the analytical sample vs. rest of cohort**

|  | Analytical sample (n = 192,825) | Rest of cohort (n = 309,394) |
| --- | --- | --- |
| **Age (years)** | 56.2 (7.9) | 56.7 (8.2) |
| **Sex (%)** |  |  |
| Females | 105880 (54.9) | 167331 (54.1) |
| Males | 86945 (45.1) | 142063 (45.9) |
| **BMI (kg/m^2^)** |  |  |
| < 25 | 72413 (37.6) | 92509 (30.2) |
| 25 – 29.9 | 79648 (41.3) | 132352 (43.1) |
| > 30 | 40233 (20.9) | 81959 (26.7) |
| **Ethnicity (%)** |  |  |
| White | 184080 (95.5) | 288280 (93.6) |
| Non-white | 7817 (4.1) | 19819 (6.4) |
| Mixed | 895 (0.5) | 1437 (0.5) |
| South Asian | 2670 (1.4) | 7202 (2.3) |
| Black | 2317 (1.2) | 5731 (1.9) |
| Chinese | 548 (0.3) | 1023 (0.3) |
| Other | 1387 (0.7) | 3165 (1.0) |
| Prefer not to say | 312 (0.2) | 1261 (0.4) |
| **Education (%)** |  |  |
| Higher | 111484 (57.8) | 121935 (40) |
| Vocational | 22432 (11.6) | 40525 (13.3) |
| Upper secondary | 11970 (6.2) | 15245 (5) |
| Lower secondary | 29094 (15.1) | 54164 (17.8) |
| None/prefer not to say | 17769 (9.2) | 72962 (23.9) |
| **Socioeconomic status (%)** |  |  |
| 1 (least deprived) | 41463 (21.5) | 59829 (19.4) |
| 2-4 | 119148 (61.8) | 181007 (58.6) |
| 5 (most deprived) | 31980 (16.6) | 68166 (22.1) |

**Table S3** – Starchy carbohydrate intake by socio-demographic factors

|  | **Total score** | **P-value** |
| --- | --- | --- |
| **All participants** | 3.15 ± 1.83 |  |
| **Age** |  | **< 0.001** |
| Younger (≤ 57 years) | 3.07 ± 1.87 |  |
| Older (> 57 years) | 3.24 ± 1.80 |  |
| **Sex** |  | **< 0.001** |
| Female | 3.29 ± 1.79 |  |
| Male | 2.98 ± 1.87 |  |
| **BMI** |  | **<0.001** |
| < 25 | 3.28 ± 1.79 |  |
| 25 – 29.9 | 3.07 ± 1.85 |  |
| > 30 | 3.10 ± 1.87 |  |
| **Ethnicity** |  | **<0.001** |
| White | 3.18 ± 1.82 |  |
| Non-white | 2.62 ± 1.98 |  |
| Mixed | 2.98 ± 1.89 |  |
| South Asian | 2.50 ± 1.98 |  |
| Black | 2.51 ± 2.02 |  |
| Chinese | 2.64 ± 1.93 |  |
| Other | 2.78 ± 1.93 |  |
| Prefer not to say | 2.97 ± 1.85 |  |
| **Education** |  | **< 0.001** |
| Higher | 3.15 ± 1.83 |  |
| Vocational | 3.11 ± 1.85 |  |
| Upper secondary | 3.11 ± 1.85 |  |
| Lower secondary | 3.16 ± 1.83 |  |
| None/prefer not to say | 3.22 ± 1.85 |  |
| **Socio-economic status** |  | **<0.001** |
| 1 (least deprived) | 3.19 ± 1.81 |  |
| 2-4 | 3.17 ± 1.83 |  |
| 5 (most deprived) | 3.04 ± 1.89 |  |

*P* value for ethnicity in relation to white vs non white participants

**Table S4** – Wholegrain intake by socio-demographic factors

|  | **Total score** | **P-value** |
| --- | --- | --- |
| **All participants** | 1.33 ± 1.87 |  |
| **Age** |  | **< 0.001** |
| Younger (≤ 57 years) | 1.22 ± 1.83 |  |
| Older (> 57 years) | 1.44 ± 1.92 |  |
| **Sex** |  | **< 0.001** |
| Female | 1.14 ± 1.75 |  |
| Male | 1.57 ± 2.00 |  |
| **BMI** |  | **<0.001** |
| < 25 | 1.48 ± 1.93 |  |
| 25 – 29.9 | 1.30 ± 1.86 |  |
| > 30 | 1.14 ± 1.79 |  |
| **Ethnicity** |  | **<0.001** |
| White | 1.35 ± 1.88 |  |
| Non-white | 1.08 ± 1.78 |  |
| Mixed | 1.24 ± 1.88 |  |
| South Asian | 1.08 ± 1.77 |  |
| Black | 1.06 ± 1.79 |  |
| Chinese | 0.87 ± 1.64 |  |
| Other | 1.07 ± 1.76 |  |
| Prefer not to say | 1.31 ± 1.85 |  |
| **Education** |  | **<0.001** |
| Higher | 1.42 ± 1.91 |  |
| Vocational | 1.25 ± 1.85 |  |
| Upper secondary | 1.28 ± 1.84 |  |
| Lower secondary | 1.14 ± 1.78 |  |
| None/prefer not to say | 1.22 ± 1.84 |  |
| **Socio-economic status** |  | **<0.001** |
| 1 (least deprived) | 1.37 ± 1.88 |  |
| 2-4 | 1.34 ± 1.87 |  |
| 5 (most deprived) | 1.28 ± 1.88 |  |

*P* value for ethnicity in relation to white vs non white participants

**Table S5** – Red and processed meat intake by socio-demographic factors

|  | **Total score** | **P-value** |
| --- | --- | --- |
| **All participants** | 3.65 ± 1.98 |  |
| **Age** |  | 0.268 |
| Younger (≤ 57 years) | 3.66 ± 1.99 |  |
| Older (> 57 years) | 3.65 ± 1.97 |  |
| **Sex** |  | **<0.001** |
| Female | 3.87 ± 1.85 |  |
| Male | 3.39 ± 2.11 |  |
| **BMI** |  | **<0.001** |
| < 25 | 3.90 ± 1.84 |  |
| 25 – 29.9 | 3.59 ± 2.01 |  |
| > 30 | 3.35 ± 2.12 |  |
| **Ethnicity** |  | **<0.001** |
| White | 3.64 ± 1.99 |  |
| Non-white | 4.13 ± 1.71 |  |
| Mixed | 3.82 ± 1.90 |  |
| South Asian | 4.45 ± 1.40 |  |
| Black | 3.98 ± 1.83 |  |
| Chinese | 3.73 ± 1.94 |  |
| Other | 4.13 ± 1.69 |  |
| Prefer not to say | 3.77 ± 1.95 |  |
| **Education** |  | **< 0.001** |
| Higher | 3.72 ± 1.95 |  |
| Vocational | 3.50 ± 2.06 |  |
| Upper secondary | 3.65 ± 1.99 |  |
| Lower secondary | 3.59 ± 2.01 |  |
| None/prefer not to say | 3.58 ± 2.00 |  |
| **Socio-economic status** |  | **<0.001** |
| 1 (least deprived) | 3.62 ± 1.99 |  |
| 2-4 | 3.65 ± 1.98 |  |
| 5 (most deprived) | 3.72 ± 1.98 |  |

*P* value for ethnicity in relation to white vs non white participants

**Table S6** – Fish intake by socio-demographic factors

|  | **Total score** | **P-value** |
| --- | --- | --- |
| **All participants** | 1.53 ± 1.95 |  |
| **Age** |  | **< 0.001** |
| Younger (≤ 57 years) | 1.41 ± 1.90 |  |
| Older (> 57 years) | 1.64 ± 2.00 |  |
| **Sex** |  | **< 0.001** |
| Female | 1.60 ± 1.98 |  |
| Male | 1.44 ± 1.92 |  |
| **BMI** |  | **< 0.001** |
| < 25 | 1.67 ± 2.01 |  |
| 25 – 29.9 | 1.49 ± 1.94 |  |
| > 30 | 1.34 ± 1.85 |  |
| **Ethnicity** |  | **<0.001** |
| White | 1.53 ± 1.96 |  |
| Non-white | 1.38 ± 1.92 |  |
| Mixed | 1.42 ± 1.90 |  |
| South Asian | 0.99 ± 1.68 |  |
| Black | 1.54 ± 2.00 |  |
| Chinese | 1.99 ± 2.05 |  |
| Other | 1.60 ± 2.02 |  |
| Prefer not to say | 1.43 ± 1.93 |  |
| **Education** |  | **< 0.001** |
| Higher | 1.66 ± 2.00 |  |
| Vocational | 1.32 ± 1.85 |  |
| Upper secondary | 1.56 ± 1.96 |  |
| Lower secondary | 1.34 ± 1.86 |  |
| None/prefer not to say | 1.21 ± 1.80 |  |
| **Socio-economic status** |  | **< 0.001** |
| 1 (least deprived) | 1.60 ± 1.98 |  |
| 2-4 | 1.52 ± 1.95 |  |
| 5 (most deprived) | 1.45 ± 1.92 |  |

*P* value for ethnicity in relation to white vs non white participants

**Table S7** – White meat intake by socio-demographic factors

|  | **Total score** | **P-value** |
| --- | --- | --- |
| **All participants** | 2.00 ± 2.44 |  |
| **Age** |  | **< 0.001** |
| Younger (≤ 57 years) | 2.07 ± 2.46 |  |
| Older (> 57 years) | 1.94 ± 2.43 |  |
| **Sex** |  | 0.95 |
| Female | 2.01 ± 2.44 |  |
| Male | 2.01 ± 2.44 |  |
| **BMI** |  | **< 0.001** |
| < 25 | 1.95 ± 2.43 |  |
| 25 – 29.9 | 2.03 ± 2.45 |  |
| > 30 | 2.06 ± 2.44 |  |
| **Ethnicity** |  | **0.64** |
| White | 2.01 ± 2.44 |  |
| Non-white | 2.02 ± 2.45 |  |
| Mixed | 1.95 ± 2.44 |  |
| South Asian | 1.69 ± 2.36 |  |
| Black | 2.33 ± 2.49 |  |
| Chinese | 2/33 ± 2.49 |  |
| Other | 2.05 ± 2.46 |  |
| Prefer not to say | 1.82 ± 2.40 |  |
| **Education** |  | **< 0.001** |
| Higher | 2.01 ± 2.44 |  |
| Vocational | 2.09 ± 2.46 |  |
| Upper secondary | 2.09 ± 2.46 |  |
| Lower secondary | 2.03 ± 2.45 |  |
| None/prefer not to say | 1.80 ± 2.40 |  |
| **Socio-economic status** |  | **< 0.001** |
| 1 (least deprived) | 2.08 ± 2.46 |  |
| 2-4 | 2.01 ± 2.44 |  |
| 5 (most deprived) | 1.89 ± 2.42 |  |

*P* value for ethnicity in relation to white vs non white participants

**Table S8** – Fruit and vegetable intake by socio-demographic factors

|  | **Total score** | **P-value** |
| --- | --- | --- |
| **All participants** | 3.25 ± 2.00 |  |
| **Age** |  | **< 0.001** |
| Younger (≤ 57 years) | 3.05 ± 2.06 |  |
| Older (> 57 years) | 3.44 ± 1.93 |  |
| **Sex** |  | **< 0.001** |
| Female | 3.52 ± 1.91 |  |
| Male | 2.91 ± 2.07 |  |
| **BMI** |  | **< 0.001** |
| < 25 | 3.43 ± 1.93 |  |
| 25 – 29.9 | 3.18 ± 2.02 |  |
| > 30 | 3.05 ± 2.08 |  |
| **Ethnicity** |  | **<0.001** |
| White | 3.26 ± 2.00 |  |
| Non-white | 3.01 ± 2.10 |  |
| Mixed | 3.13 ± 2.07 |  |
| South Asian | 2.91 ± 2.11 |  |
| Black | 2.81 ± 2.16 |  |
| Chinese | 3.28 ± 1.96 |  |
| Other | 3.36 ± 2.02 |  |
| Prefer not to say | 3.12 ± 2.04 |  |
| **Education** |  | **< 0.001** |
| Higher | 3.44 ± 1.92 |  |
| Vocational | 2.94 ± 2.09 |  |
| Upper secondary | 3.19 ± 2.02 |  |
| Lower secondary | 2.99 ± 2.08 |  |
| None/prefer not to say | 2.86 ± 2.12 |  |
| **Socio-economic status** |  | **< 0.001** |
| 1 (least deprived) | 3.33 ± 1.96 |  |
| 2-4 | 3.27 ± 2.00 |  |
| 5 (most deprived) | 3.07 ± 2.08 |  |

*P* value for ethnicity in relation to white vs non white participants

**Table S9** – Dairy intake by socio-demographic factors

|  | **Total score** | **P-value** |
| --- | --- | --- |
| **All participants** | 4.21 ± 1.52 |  |
| **Age** |  | **< 0.001** |
| Younger (≤ 57 years) | 4.24 ± 1.50 |  |
| Older (> 57 years) | 4.18 ± 1.53 |  |
| **Sex** |  | **< 0.001** |
| Female | 4.20 ± 1.52 |  |
| Male | 4.22 ± 1.51 |  |
| **BMI** |  | 0.712 |
| < 25 | 4.21 ± 1.51 |  |
| 25 – 29.9 | 4.21 ± 1.51 |  |
| > 30 | 4.20 ± 1.54 |  |
| **Ethnicity** |  | **<0.001** |
| White | 4.20 ± 1.52 |  |
| Non-white | 4.49 ± 1.31 |  |
| Mixed | 4.31 ± 1.48 |  |
| South Asian | 4.49 ± 1.30 |  |
| Black | 4.60 ± 1.19 |  |
| Chinese | 4.75 ± 0.93 |  |
| Other | 4.34 ± 1.47 |  |
| Prefer not to say | 4.22 ± 1.54 |  |
| **Education** |  | **< 0.001** |
| Higher | 4.18 ± 1.54 |  |
| Vocational | 4.24 ± 1.50 |  |
| Upper secondary | 4.24 ± 1.50 |  |
| Lower secondary | 4.25 ± 1.48 |  |
| None/prefer not to say | 4.26 ± 1.48 |  |
| **Socio-economic status** |  | **< 0.001** |
| 1 (least deprived) | 4.18 ± 1.53 |  |
| 2-4 | 4.21 ± 1.51 |  |
| 5 (most deprived) | 4.24 ± 1.52 |  |

*P* value for ethnicity in relation to white vs non white participants

**Table S10** – Beans and pulses intake by socio-demographic factors

|  | **Total score** | **P-value** |
| --- | --- | --- |
| **All participants** | 2.02 ± 2.33 |  |
| **Age** |  | **< 0.001** |
| Younger (≤ 57 years) | 1.94 ± 2.32 |  |
| Older (> 57 years) | 2.11 ± 2.35 |  |
| **Sex** |  | **0.02** |
| Female | 2.01 ± 2.33 |  |
| Male | 2.04 ± 2.34 |  |
| **BMI** |  | **< 0.001** |
| < 25 | 2.07 ± 2.33 |  |
| 25 – 29.9 | 2.02 ± 2.34 |  |
| > 30 | 1.97 ± 2.33 |  |
| **Ethnicity** |  | **< 0.001** |
| White | 2.03 ± 2.34 |  |
| Non-white | 1.88 ± 2.32 |  |
| Mixed | 1.95 ± 2.32 |  |
| South Asian | 2.26 ± 2.39 |  |
| Black | 1.46 ± 2.18 |  |
| Chinese | 1.83 ± 2.28 |  |
| Other | 1.80 ± 2.29 |  |
| Prefer not to say | 2.02 ± 2.34 |  |
| **Education** |  | **< 0.001** |
| Higher | 2.08 ± 2.34 |  |
| Vocational | 1.96 ± 2.33 |  |
| Upper secondary | 1.98 ± 2.33 |  |
| Lower secondary | 1.96 ± 2.33 |  |
| None/prefer not to say | 1.91 ± 2.33 |  |
| **Socio-economic status** |  | **< 0.001** |
| 1 (least deprived) | 2.06 ± 2.34 |  |
| 2-4 | 2.04 ± 2.34 |  |
| 5 (most deprived) | 1.94 ± 2.33 |  |

*P* value for ethnicity in relation to white vs non white participants

**Table S11** – Nuts intake by socio-demographic factors

|  | **Total score** | **P-value** |
| --- | --- | --- |
| **All participants** | 1.00 ± 1.99 |  |
| **Age** |  | **< 0.001** |
| Younger (≤ 57 years) | 0.95 ± 1.95 |  |
| Older (> 57 years) | 1.05 ± 2.02 |  |
| **Sex** |  | **< 0.001** |
| Female | 1.04 ± 2.01 |  |
| Male | 0.96 ± 1.96 |  |
| **BMI** |  | **< 0.001** |
| < 25 | 1.17 ± 0.97 |  |
| 25 – 29.9 | 0.97 ± 1.96 |  |
| > 30 | 0.76 ± 1.78 |  |
| **Ethnicity** |  | **<0.001** |
| White | 0.99 ± 1.98 |  |
| Non-white | 1.28 ± 2.17 |  |
| Mixed | 1.19 ± 2.12 |  |
| South Asian | 1.43 ± 2.25 |  |
| Black | 1.03 ± 2.02 |  |
| Chinese | 1.77 ± 2.37 |  |
| Other | 1.28 ± 2.16 |  |
| Prefer not to say | 0.98 ± 1.97 |  |
| **Education** |  | **< 0.001** |
| Higher | 1.11 ± 2.06 |  |
| Vocational | 0.85 ± 1.87 |  |
| Upper secondary | 1.02 ± 2.00 |  |
| Lower secondary | 0.87 ± 1.88 |  |
| None/prefer not to say | 0.73 ± 1.75 |  |
| **Socio-economic status** |  | **0.387** |
| 1 (least deprived) | 1.01 ± 1.99 |  |
| 2-4 | 1.00 ± 1.93 |  |
| 5 (most deprived) | 1.01 ± 2.00 |  |

*P* value for ethnicity in relation to white vs non white participants

**Table S12** – Egg intake by socio-demographic factors

|  | **Total score** | **P-value** |
| --- | --- | --- |
| **All participants** | 1.64 ± 2.35 |  |
| **Age** |  | **< 0.001** |
| Younger (≤ 57 years) | 1.54 ± 2.31 |  |
| Older (> 57 years) | 1.74 ± 2.38 |  |
| **Sex** |  | 0.08 |
| Female | 1.64 ± 2.35 |  |
| Male | 1.63 ± 2.34 |  |
| **BMI** |  | **< 0.001** |
| < 25 | 1.58 ± 2.32 |  |
| 25 – 29.9 | 1.64 ± 2.34 |  |
| > 30 | 1.74 ± 2.38 |  |
| **Ethnicity** |  | 0.74 |
| White | 1.64 ± 2.35 |  |
| Non-white | 1.65 ± 2.35 |  |
| Mixed | 1.57 ± 2.32 |  |
| South Asian | 1.49 ± 2.29 |  |
| Black | 1.54 ± 2.31 |  |
| Chinese | 2.41 ± 2.50 |  |
| Other | 1.87 ± 2.42 |  |
| Prefer not to say | 1.73 ± 2.38 |  |
| **Education** |  | **< 0.001** |
| Higher | 1.65 ± 2.35 |  |
| Vocational | 1.60 ± 2.33 |  |
| Upper secondary | 1.66 ± 2.35 |  |
| Lower secondary | 1.60 ± 2.33 |  |
| None/prefer not to say | 1.66 ± 2.36 |  |
| **Socio-economic status** |  | **< 0.001** |
| 1 (least deprived) | 1.60 ± 2.33 |  |
| 2-4 | 1.63 ± 2.34 |  |
| 5 (most deprived) | 1.72 ± 2.37 |  |

*P* value for ethnicity in relation to white vs non white participants

**Table S13** – Discretionary foods intake by socio-demographic factors

|  | **Total score** | **P-value** |
| --- | --- | --- |
| **All participants** | 1.40 ± 2.18 |  |
| **Age** |  | **< 0.001** |
| Younger (≤ 57 years) | 1.37 ± 2.17 |  |
| Older (> 57 years) | 1.43 ± 2.20 |  |
| **Sex** |  | **< 0.001** |
| Female | 1.58 ± 2.25 |  |
| Male | 1.18 ± 2.08 |  |
| **BMI** |  | **< 0.001** |
| < 25 | 1.41 ± 2.18 |  |
| 25 – 29.9 | 1.37 ± 2.17 |  |
| > 30 | 1.44 ± 2.22 |  |
| **Ethnicity** |  | **<0.001** |
| White | 1.40 ± 2.18 |  |
| Non-white | 1.33 ± 2.16 |  |
| Mixed | 1.26 ± 2.12 |  |
| South Asian | 1.28 ± 2.14 |  |
| Black | 1.14 ± 2.06 |  |
| Chinese | 1.79 ± 2.33 |  |
| Other | 1.60 ± 2.28 |  |
| Prefer not to say | 1.34 ± 2.18 |  |
| **Education** |  | **< 0.001** |
| Higher | 1.50 ± 2.22 |  |
| Vocational | 1.20 ± 2.08 |  |
| Upper secondary | 1.40 ± 2.18 |  |
| Lower secondary | 1.27 ± 2.12 |  |
| None/prefer not to say | 1.27 ± 2.13 |  |
| **Socio-economic status** |  | **< 0.001** |
| 1 (least deprived) | 1.38 ± 2.17 |  |
| 2-4 | 1.39 ± 2.18 |  |
| 5 (most deprived) | 1.45 ± 2.21 |  |

*P* value for ethnicity in relation to white vs non white participants

**Table S14** – Fluid intake by socio-demographic factors

|  | **Total score** | **P-value** |
| --- | --- | --- |
| **All participants** | 3.53 ± 1.76 |  |
| **Age** |  | **< 0.001** |
| Younger (≤ 57 years) | 3.59 ± 1.75 |  |
| Older (> 57 years) | 3.47 ± 1.77 |  |
| **Sex** |  | **< 0.001** |
| Female | 3.80 ± 1.61 |  |
| Male | 3.20 ± 1.88 |  |
| **BMI** |  | **< 0.001** |
| < 25 | 1.58 ± 2.32 |  |
| 25 – 29.9 | 1.64 ± 2.34 |  |
| > 30 | 1.74 ± 2.38 |  |
| **Ethnicity** |  | **< 0.001** |
| White | 3.55 ± 1.76 |  |
| Non-white | 3.18 ± 1.90 |  |
| Mixed | 3.38 ± 1.86 |  |
| South Asian | 3.36 ± 1.82 |  |
| Black | 2.68 ± 1.99 |  |
| Chinese | 3.50 ± 1.78 |  |
| Other | 3.39 ± 1.80 |  |
| Prefer not to say | 3.32 ± 1.88 |  |
| **Education** |  | **< 0.001** |
| Higher | 1.65 ± 2.35 |  |
| Vocational | 1.60 ± 2.33 |  |
| Upper secondary | 1.66 ± 2.35 |  |
| Lower secondary | 1.60 ± 2.33 |  |
| None/prefer not to say | 1.66 ± 2.36 |  |
| **Socio-economic status** |  | **< 0.001** |
| 1 (least deprived) | 1.60 ± 2.33 |  |
| 2-4 | 1.63 ± 2.34 |  |
| 5 (most deprived) | 1.72 ± 2.37 |  |

*P* value for ethnicity in relation to white vs non white participants

**Table S15**. Proportion of participants achieving adherence to individual components (i.e. 5 points) of the Eatwell guide.

|  | **Participants achieving full adherence (N (%))** |
| --- | --- |
| **Starchy carbohydrates** | 75574 (39.2) |
| **Wholegrains** | 29470 (15.3) |
| **Red and processed meat** | 108600 (56.3) |
| **Fish** | 34432 (17.9) |
| **White meat** | 76756 (39.8) |
| **Fruit and vegetables** | 93877 (48.7) |
| **Dairy** | 139460 (72.3) |
| **Beans and pulses** | 68868 (35.7) |
| **Nuts** | 37587 (19.5) |
| **Eggs** | 63065 (32.7) |
| **Discretionary foods** | 49847 (25.9) |
| **Fluid** | 91115 (47.3) |

**Table S16**. Sensitivity analysis demonstrating adherence to the Eatwell guide by socio-demographic characteristics, only including those participants who completed a minimum of two dietary reports

|  | **Total score** | **P-value** |
| --- | --- | --- |
| **All participants** |  |  |
| **Age** |  | **<0.001** |
| Younger (≤ 57 years) | 29.61 ± 8.24 |  |
| Older (> 57 years) | 31.01 ± 8.26 |  |
| **Sex** |  | **<0.001** |
| Female | 31.25 ± 8.03 |  |
| Male | 29.10 ± 8.43 |  |
| **BMI** |  | **<0.001** |
| < 25 | 31.19 ± 8.29 |  |
| 25 – 29.9 | 29.90 ± 8.25 |  |
| > 30 | 29.34 ± 8.14 |  |
| **Ethnicity** |  | 0.93 |
| White | 30.3 ± 8.3 |  |
| Non-white | 30.3 ± 8.4 |  |
| Mixed | 29.8 ± 8.6 |  |
| South Asian | 30.2 ± 7.8 |  |
| Black | 29.0 ± 8.8 |  |
| Chinese | 32.4 ± 8.7 |  |
| Other | 31.5 ± 8.4 |  |
| Prefer not to say | 29.6 ± 8.2 |  |
| **Education** |  | **<0.001** |
| Higher | 30.88 ± 8.22 |  |
| Vocational | 29.04 ± 8.27 |  |
| Upper secondary | 30.18 ± 8.39 |  |
| Lower secondary | 29.22 ± 8.23 |  |
| None/prefer not to say | 29.27 ± 8.25 |  |
| **Socio-economic status** |  | **<0.001** |
| 1 (least deprived) | 30.52 ± 8.17 |  |
| 2-4 | 30.38 ± 8.25 |  |
| 5 (most deprived) | 29.98 ± 8.53 |  |

*P* value for ethnicity in relation to white vs non white participants

**Table S17**. Sensitivity analysis demonstrating adherence to the Eatwell guide by socio-demographic characteristics, excluding dietary reports with extreme energy intakes

|  | **Total score** | **P-value** |
| --- | --- | --- |
| **All participants** |  |  |
| **Age** |  | **<0.001** |
| Younger (≤ 57 years) | 28.09 ± 8.34 |  |
| Older (> 57 years) | 29.33 ± 8.37 |  |
| **Sex** |  | **<0.001** |
| Female | 29.68 ± 8.13 |  |
| Male | 27.51 ± 8.51 |  |
| **BMI** |  | **<0.001** |
| < 25 | 29.67 ± 8.44 |  |
| 25 – 29.9 | 28.32 ± 8.32 |  |
| > 30 | 27.75 ± 8.19 |  |
| **Ethnicity** |  | **<0.001** |
| White | 28.76 ± 8.40 |  |
| Non-white | 28.05 ± 8.51 |  |
| Mixed | 28.18 ± 8.83 |  |
| South Asian | 27.91 ± 8.05 |  |
| Black | 26.72 ± 8.66 |  |
| Chinese | 30.88 ± 8.66 |  |
| Other | 29.28 ± 8.48 |  |
| Prefer not to say | 28.01 ± 8.31 |  |
| **Education** |  | **<0.001** |
| Higher | 29.57 ± 8.30 |  |
| Vocational | 27.37 ± 8.31 |  |
| Upper secondary | 28.67 ± 8.48 |  |
| Lower secondary | 27.55 ± 8.28 |  |
| None/prefer not to say | 26.87 ± 8.26 |  |
| **Socio-economic status** |  | **<0.001** |
| 1 (least deprived) | 29.02 ± 8.27 |  |
| 2-4 | 28.74 ± 8.33 |  |
| 5 (most deprived) | 28.16 ± 8.63 |  |

**Table S18.** Sensitivity analysis of EWG adherence score by socio-demographic factors when sequentially removing one component of EWG

|  | **Full score** | **Minus starchy carbohy-drate** | **Minus wholegrains** | **Minus red and processed meat** | **Minus fish** | **Minus white meat** | **Minus fruit and veg** | **Minus dairy** | **Minus beans and pulses** | **Minus nuts** | **Minus egg** | **Minus discreti-onary food** | **Minus fluid** |
| --- | --- | --- | --- | --- | --- | --- | --- | --- | --- | --- | --- | --- | --- |
| **Age** | **< 0.001** | **< 0.001** | **< 0.001** | **< 0.001** | **< 0.001** | **< 0.001** | **< 0.001** | **< 0.001** | **< 0.001** | **< 0.001** | **< 0.001** | **< 0.001** | **< 0.001** |
| **Sex** | **< 0.001** | **< 0.001** | **< 0.001** | **< 0.001** | **< 0.001** | **< 0.001** | **< 0.001** | **< 0.001** | **< 0.001** | **< 0.001** | **< 0.001** | **< 0.001** | **< 0.001** |
| **BMI** | **< 0.001** | **< 0.001** | **< 0.001** | **< 0.001** | **< 0.001** | **< 0.001** | **< 0.001** | **< 0.001** | **< 0.001** | **< 0.001** | **< 0.001** | **< 0.001** | **< 0.001** |
| **Ethnicity** | **< 0.001** | **0.08** | **< 0.001** | **< 0.001** | **< 0.001** | **< 0.001** | **< 0.001** | **< 0.001** | **< 0.001** | **< 0.001** | **< 0.001** | **< 0.001** | **< 0.001** |
| **Education** | **< 0.001** | **< 0.001** | **< 0.001** | **< 0.001** | **< 0.001** | **< 0.001** | **< 0.001** | **< 0.001** | **< 0.001** | **< 0.001** | **< 0.001** | **< 0.001** | **< 0.001** |
| **Socio-economic status** | **< 0.001** | **< 0.001** | **< 0.001** | **< 0.001** | **< 0.001** | **< 0.001** | **< 0.001** | **< 0.001** | **< 0.001** | **< 0.001** | **< 0.001** | **< 0.001** | **< 0.001** |

Values are *p* values
